# Aflibercept for long-term treatment of diabetic macular edema and proliferative diabetic retinopathy: a meta-analysis

**DOI:** 10.3389/fendo.2023.1144422

**Published:** 2023-05-16

**Authors:** Xiao Xie, Chao Lian, Zhiping Zhang, Meng Feng, Wenqi Wang, Xiaomeng Yuan, Yanmei Shi, Tingting Liu

**Affiliations:** ^1^ First Clinical Medical College, Shandong University of Traditional Chinese Medicine, Jinan, China; ^2^ Eye Hospital of Shandong First Medical University, Shandong Eye Hospital, Jinan, China; ^3^ State Key Laboratory Cultivation Base, Shandong Provincial Key Laboratory of Ophthalmology, Shandong Eye Institute, Shandong First Medical University & Shandong Academy of Medical Sciences, Qingdao, China; ^4^ Chinese Medicine College, Shandong University of Traditional Chinese Medicine, Jinan, China; ^5^ Laboratory Department, Affiliated Hospital of Shanxi University of Chinese Medicine, Xianyang, China

**Keywords:** diabetic macular edema, proliferative diabetic retinopathy, aflibercept, meta-analysis, anti-vascular endothelial growth factor, focal/grid laser photocoagulation, panretinal photocoagulation

## Abstract

**Purpose:**

This meta-analysis compared the long-term (12 months or 24 months) efficacy and safety of intravitreal aflibercept injection (IAI) for diabetic macular edema (DME) and proliferative diabetic retinopathy (PDR).

**Methods:**

We selected 16 randomized controlled trials (RCTs) performed after 2015 that had a minimum of 12 months and up to 24 months of treatment and conducted a meta-analysis with Review Manager version 5.3. Visual acuity (VA), central subfield thickness (CST) and adverse events were the outcomes selected for evaluation from the eligible studies.

**Results:**

Based on 16 RCTs, we evaluated a total of 7125 patients. For PDR and severe DME with poor baseline vision, after a minimum of 12 months and up to 24 months of treatment, the aflibercept treatment group obtained better VA improvement than the focal/grid laser photocoagulation treatment group (MD=13.30; 95%CI: 13.01~13.58; *P*<0.001) or other treatments (ranibizumab, focal/grid laser photocoagulation, PRP, et al.) group (MD=1.10; 95%CI: 1.05~1.16; *P*<0.001). In addition, the aflibercept treatment group got higher CST reduction than the focal/grid laser photocoagulation treatment (MD=-33.76; 95%CI: -45.53 ~ -21.99; *P*<0.001) or other treatments (ranibizumab, focal/grid laser photocoagulation, et al.) group (MD=-33.76; 95%CI: -45.53 ~ -21.99; *P*<0.001). There was no significant difference in the overall incidence of ocular and non-ocular adverse events in each treatment group.

**Conclusions:**

This meta-analysis showed that the advantages of IAI are obvious in the management of DME and PDR with poor baseline vision for long-term observation (a minimum of 12 months and up to 24 months) with both VA improvement and CST reduction. Applied IAI separately trended to be more effective than panretinal photocoagulation separately in VA improvement for PDR. More parameters should be required to assess functional and anatomic outcomes.

## 1 Introduction

Diabetic retinopathy (DR) has become an increasingly common microvascular complication of diabetes that affects visual health. Proliferative diabetic retinopathy (PDR) and severe diabetic macular edema (DME), especially center-involved DME (CI-DME), have become the most common causes of visual loss among working population (1–3).

PDR and DME have been managed by panretinal laser photocoagulation (PRP) and focal/grid laser photocoagulation for the past 40 years (4, 5). Vascular endothelial growth factor (VEGF), as a “real killer” that seriously threatens vision, plays an important role in the improvement of diabetic retinal vascular permeability and is an important contributor to both vascular leakage and new blood vessel growth (6, 7). To date, many researchers have conducted systematic reviews and standard meta-analyses of anti-VEGF treatments which have been recognized as novel approaches for visual impairment of DR (8, 9). However, these existing meta-analysis did not include direct and indirect comparisons of the long-term observation (a minimum of 12 months and up to 24 months) of aflibercept and focal/grid laser photocoagulation or other treatments respectively in patients with DME, or afliberceptand panretinal photocoagulation (PRP) in patients with PDR. Therefore, we conducted a meta-analysis that included randomized controlled trials (RCTs) reported after 2015. This report compared the therapeutic effects of intravitreal injections of aflibercept with that of focal/grid laser photocoagulation (patients with DME), PRP (patients with PDR) or other treatments, evaluated the efficacy and safety of intravitreal aflibercept injection (IAI) in the management of DME and PDR for long-term observation (a minimum of 12 months and up to 24 months).

## 2 Materials and methods

### 2.1 Search strategy

We systematically searched and identified relevant trials and literature from the PubMed, Embase, and Web of Science databases, as well as the Cochrane Central Register of Controlled Trials, and the publication time is until January 2023. The scope of the search was restricted to both English languages. The following key search points and medical keywords were used: *diabetic retinopathy, randomized controlled trials, aflibercept, anti-vascular endothelial growth factor, focal/grid laser photocoagulation, panretinal photocoagulation, diabetic macular edema, and proliferative diabetic retinopathy.* Retrospective research, reviews,case reports, letters and surveys were excluded. Visual acuity (VA), central subfield thickness (CST), and adverse events were the focus of our meta-analysis. Only anonymous online public data were used in the research, without the active participation of patients and informed consent.

### 2.2 Eligibility of studies

The RCTs that meet the following criteria were considered eligible: (1) participants over 18 years of age with type 1 or 2 diabetes; (2) participants with DME or PDR; (3) published number of patients, age, gender, and intervention details; (4) treatments of interest were intravitreal injection of aflibercept 2.0mg compared with other treatment schemes, including ranibizumab 0.3mg/0.5mg, dexamethasone 0.7mg, brolucizumab 6.0 mg, faricimab 6.0 mg, focal/grid laser photocoagulation and PDR; the treatments determined by individual researchers could be proactive (fixed), reactive (pro re nata, PRN), or proactive/reactive (treat and extend, T&E); (5) the follow-up time of these study were 12 months or more; (6) studies that provided main outcomes evaluation parameters as mean ± SD: mean change in best-corrected visual acuity (BCVA) [measure in Early Treatment Diabetic Retinopathy Study (ETDRS) letters), mean change in CST, and adverse events; (7) all included studies should be compliant with the Declaration of Helsinki and written informed consent from enrolled patients; (8) if the same research subjects were reported in different publications, only the most recent and authoritative publications with available data for targeted outcomes was included.

The exclusion criteria were as follows: (1) retrospective studies and review articles; (2) unpublished data were not adopted; (3) participants only suffered from non-proliferative diabetic retinopathy; (4) no comparison was made between aflibercept and other treatment schemes; (5) RCTs with too short follow-up time.

### 2.3 Data extraction and quality assessment

The assessment of the full-text articles and data extraction of each study was independently conducted by two authors. In case of disagreement between two authors, the third author assessed again. Assessment contents included: publication metrics (name of the first author, year of publication, location and study design, etc.), the information of the participants (diagnosis, sample size, demographic characteristics, clinical characteristics, criteria of inclusion and exclusion), the information on intervention (options/frequency of treatment, dosage of medicine, duration of follow-up), and the main information on outcomes (BCVA, CST and adverse events).

Two authors independently assessed the risk of bias of the included RCTs, including random sequence generation, allocation concealment, blinding of participants and personnel, blinding of outcome assessment, incomplete outcome data, selective outcome reporting, and other bias (10). The divergences were resolved through full discussion, with the assistance of a third author if necessary.

### 2.4 Statistical analysis

Review Manager 5.3 was used for statistical analysis. The visual evaluation parameter was BCVA, the anatomical evaluation parameter was CST, and safety indicators included systemic or ocular adverse events during the injection treatment. The fixed effect model was used for data processing (11). Continuous outcomes were estimated using the mean difference (MD) and 95% credible intervals (CIs). Dichotomous outcomes were estimated using the risk difference (RD) and 95% CIs. Forest plots were used to summarize the weighted estimates.

## 3 Results

### 3.1 Literature search

**Figure 1:**
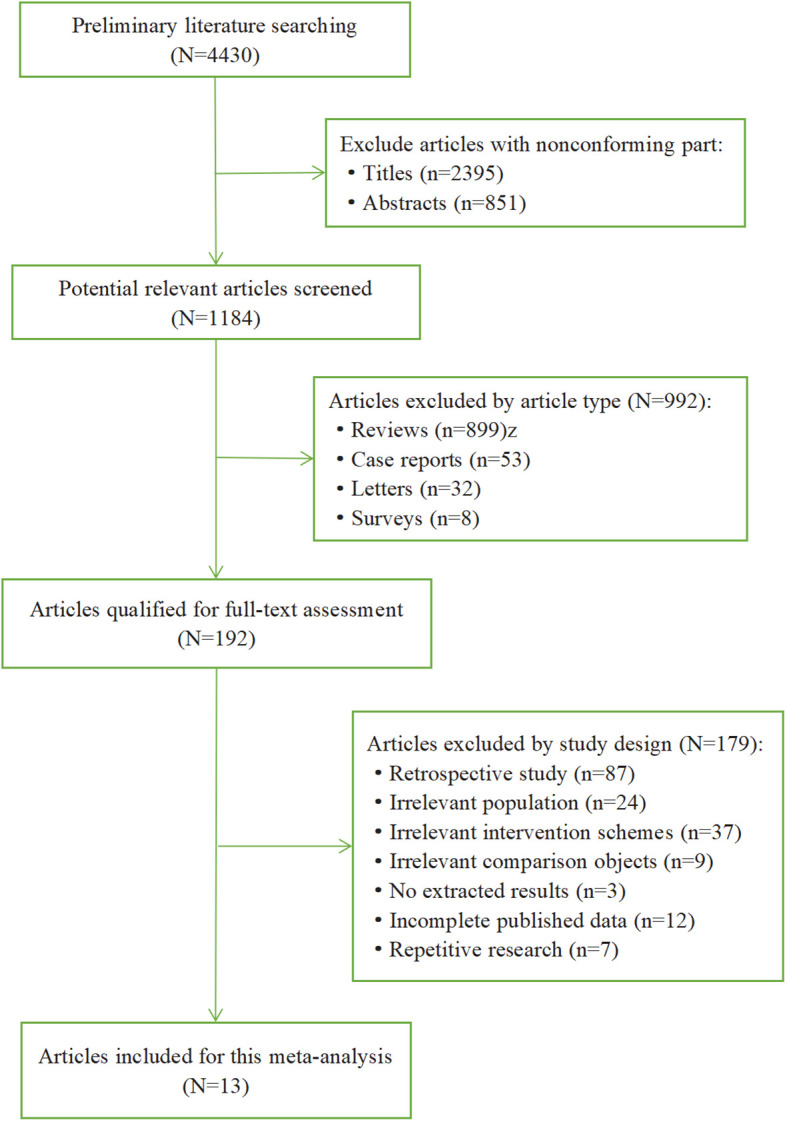
Flow chart of literature searching.

We performed a preliminary literature search through all databases and retrieved 4430 articles. The literature selection process and reasons for exclusion are summarized in **Figure 1**. First, we excluded 2395 articles with nonconforming title and 851 articles with nonconforming abstracts, and then 1184 potential relevant articles were screened out. Next, we excluded 992 articles according to the article type, including review (n=899), case reports (n=53), letters (n=32), surveys (n=8). Then there were 192 articles qualified for full-text assessment. Moreover, 179 articles were excluded by study design, including retrospective study (n=87), irrelevant population (n=24), irrelevant intervention schemes (n=37), irrelevant comparison objects (n=9), no extracted results (n=3), incomplete published data (n=12), repetitive research (n=7). Ultimately, 13 articles (16 RCTs) were included in this meta-analysis.

### 3.2 Characteristics of the included studies

**Table 1** summarizes the basic characteristics of the 16 RCTs in the 13 articles included in this meta-analysis. The study sample sizes ranged from 42 to 951 patients. The characteristics of the patients with DME or PDR were similar among the trials. The follow-up duration ranged from 12 months to 24 months. The dose of aflibercept was 2.0 mg in the aflibercept treatment groups in all included studies (12–24). Other treatments include focal/grid laser photocoagulation, PRP, vitrectomy with PRP, and intravitreal injection of ranibizumab 0.3mg/0.5mg, dexamethasone 0.7mg, brolucizumab 6.0 mg, faricimab 6.0 mg.

**Table 1:** Study characteristics of the included 16 RCTs.

	Study	Design	Disease	Numbers of participants	Gender (F/M)	Interventions details	Follow-up (months)
1	Brown VISTA 2015 (12)	VISTA	DME	461	45.5%/54.5%	Aflibercept 2.0mg	24
						Laser photocoagulation	
2	Brown VIVID 2015 (12)	VIVID	DME	404	38.2%/61.8%	Aflibercept 2.0mg	24
						Laser photocoagulation	
3	DRCR.net 2015 (13)	Protocol T	CI-DME	660	47%/53%	Aflibercept 2.0mg	12
						Ranibizumab 0.3mg	
4	Wells 2016 (14)	Protocol T	CI-DME	660	47%/53%	Aflibercept 2.0mg	24
						Ranibizumab 0.3mg	
5	Sivaprasad 2017 (15)	CLARITY	PDR	232	33.2%/66.8%	Aflibercept 2.0mg	24
						PRP	
6	Fouda 2017 (16)	RCT	DME	42	NR-no rated	Aflibercept 2.0mg	24
						Ranibizumab 0.5mg	
7	Baker 2019 (17)	RCT	CI-DME	702	38%/62%	Aflibercept 2.0mg	24
						Laser photocoagulation	
8	Ozsaygili 2019 (18)	RCT	DME	62	43.5%/56.5%	Aflibercept 2.0mg	12
						Dexamethasone 0.7mg	
9	Chen 2020 (19)	VIVID-East	DME	381	49.7%/50.3%	Aflibercept 2.0mg	12
						Laser photocoagulation	
10	Chatzirallis 2020 (20)	RCT	DME	112	45.5%/54.5%	Aflibercept 2.0mg	12
						Ranibizumab 0.5mg	
11	Antoszyk 2020 (21)	RCT	PDR	205	44%/56%	Aflibercept 2.0mg	24
						Vitrectomy with PRP	
12	Beaulieu 2021 (22)	Protocol V	CI-DME	387	37%/63%	Aflibercept 2.0mg	24
						Laser photocoagulation	
13	Brown KESTREL 2022 (23)	KESTREL	DME	566	37.3%/62.7%	Aflibercept 2.0mg	12
						Brolucizumab 6.0 mg	
14	Brown KITE 2022 (23)	KITE	DME	360	34.7%/65.3%	Aflibercept 2.0mg	12
						Brolucizumab 6.0 mg	
15	Wykof YOSEMITE 2022 (24)	YOSEMITE	DME	940	40.2%/59.8%	Aflibercept 2.0mg	12
						Faricimab 6.0 mg	
16	Wykof RHINE 2022 (24)	RHINE	DME	951	39.1%/60.9%	Aflibercept 2.0mg	12
						Faricimab 6.0 mg	

RCT, randomized controlled trial; F, female; M, male; DME, diabetic macular edema; CI-DME, center-involved diabetic macular edema; PDR, proliferative diabetic retinopathy; PRP, panretinal photocoagulation.

### 3.3 Risk of bias

**Figure 2:**
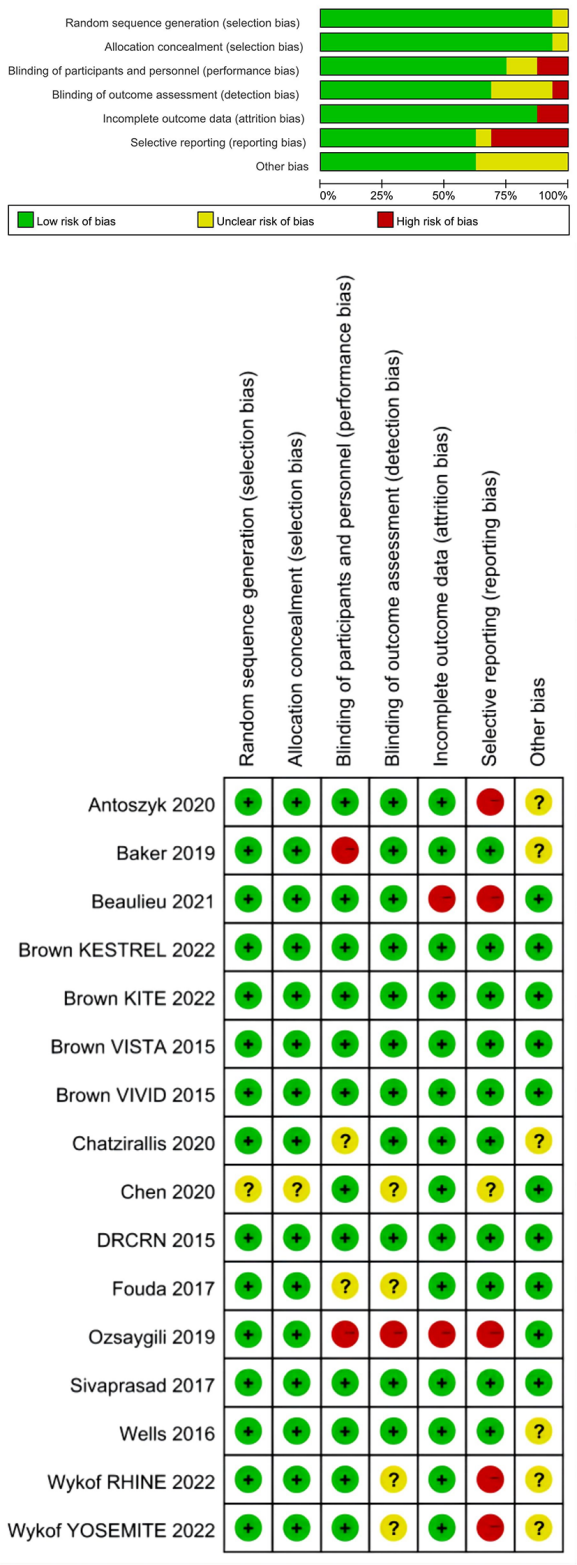
Risk of bias graph and summary for each included study.

**Figure 2** showed the risk of bias graph and summary for each included study. Fifteen studies had random sequence generation and allocation concealment. Regarding blinding of participants and personnel, 12 studies were assessed as low risk and 2 studies as high risk. Regarding blinding of outcome assessment, 11 studies were assessed as low risk and one study as high risk. Regarding incomplete outcome data, 14 studies were assessed as low risk and 2 studies as high risk. Regarding selective outcome reporting, 10 studies were assessed as low risk and 5 studies as high risk. Other studies were rated as having unclear risks.

### 3.4 Effects of interventions

#### 3.4.1 Visual acuity

**Figure 3:**
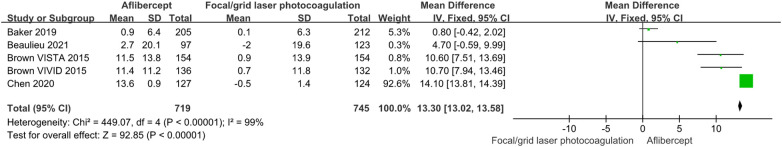
The mean changes from baseline in BCVA in the aflibercept group and focal/grid laser photocoagulation treatment group.

**Figure 4:**
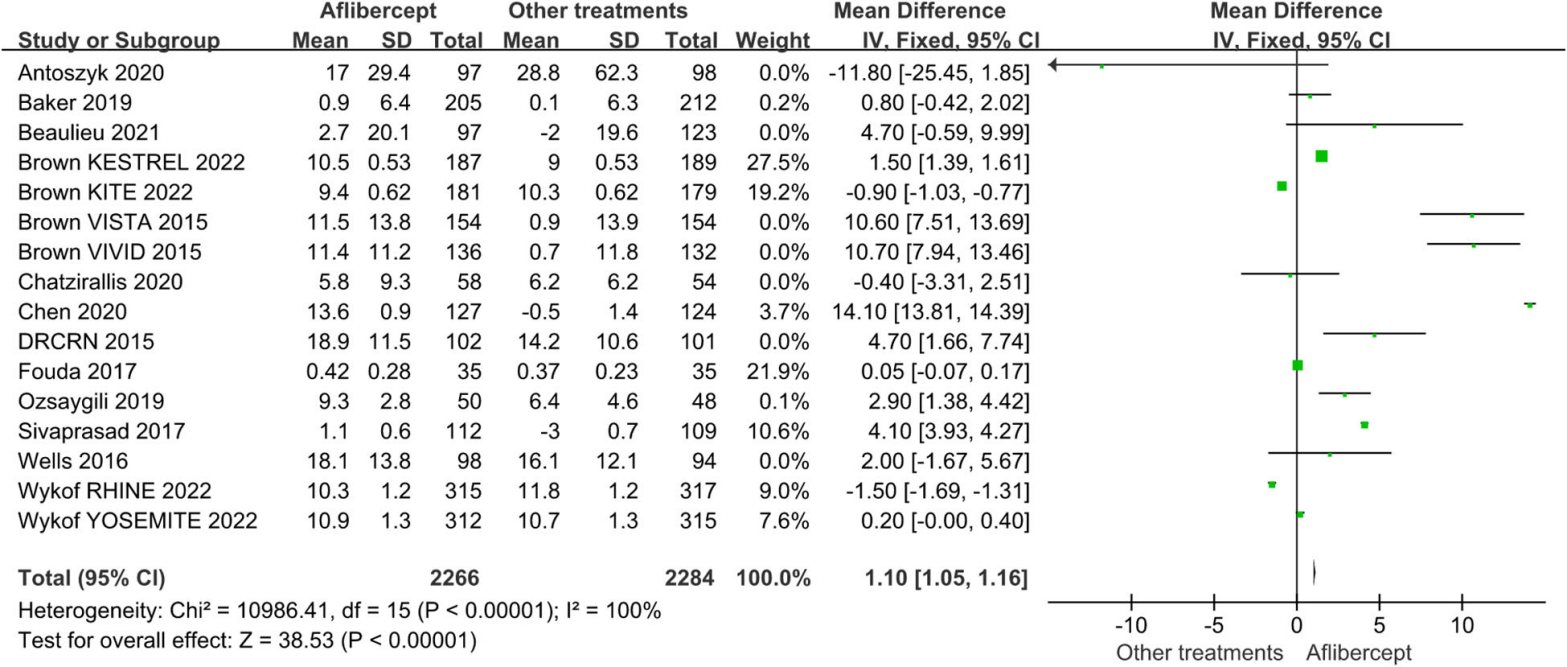
The mean changes from baseline in BCVA in the aflibercept group and other treatments group.

Because BCVA is the main visual index to judge the curative effect and progress, and CST is an important anatomical index to judge the degree of macular edema, we analyzed the data of BCVA and CST. Among these RCTs we have included, the baseline BCVA and CST did not exactly match. Therefore, we adopted the mean change in BCVA and CST as the primary outcome. **Figures 3** and **4** showed the results of the meta-analysis of the effects of intravitreal aflibercept injection on BCVA improvement. The aflibercept treatment group had significantly better BCVA improvement than the focal/grid laser photocoagulation treatment group (MD=13.30; 95%CI: 13.01~13.58; *P*<0.001) or other treatments (ranibizumab, focal/grid laser photocoagulation, PRP, et al.) group (MD=1.10; 95%CI: 1.05~1.16; *P*<0.001).

#### 3.4.2 Central subfield thickness

**Figure 5:**
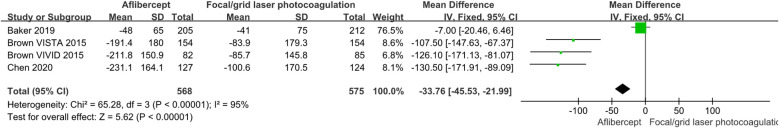
The mean changes from baseline in CST in the aflibercept group and focal/grid laser photocoagulation treatment group.

**Figure 6:**
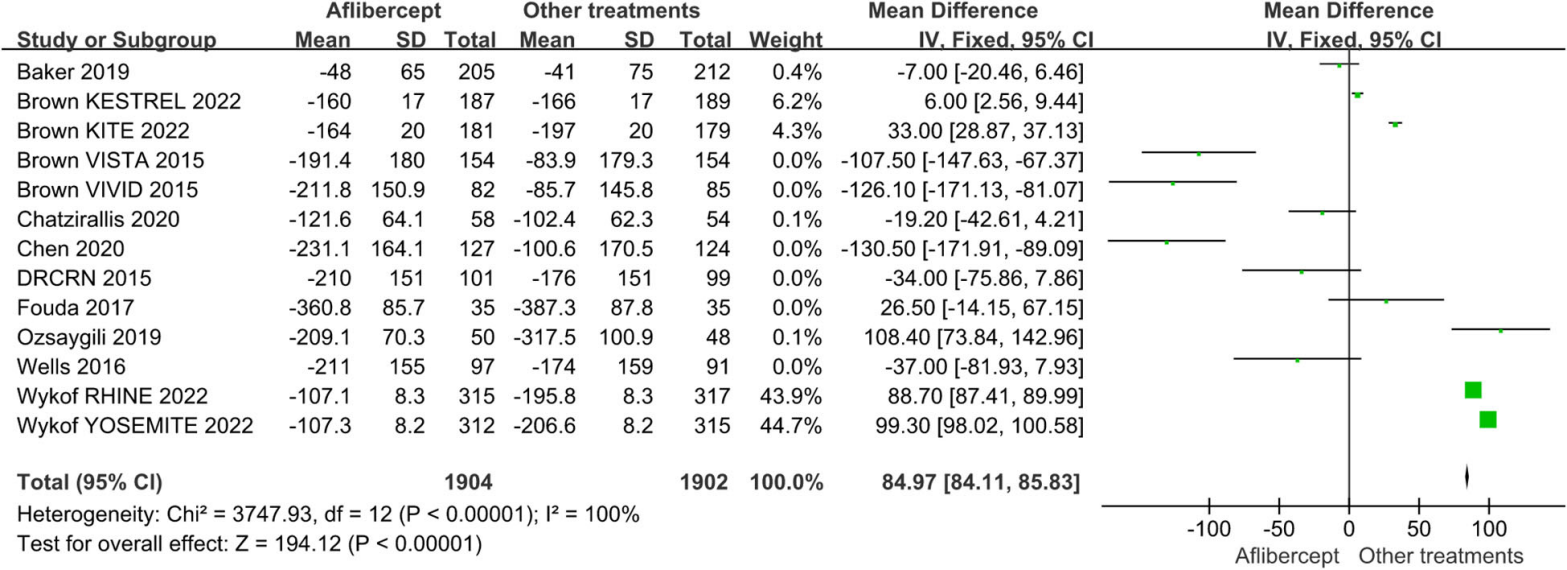
The mean changes from baseline in CST in the aflibercept group and other treatments group.

The effects of IAI in CST are shown in **Figures 5** and **6**. The aflibercept treatment group had higher CST reduction than the focal/grid laser photocoagulation treatment group (MD=-33.76; 95%CI: -45.53 ~ -21.99; *P*<0.001) or other treatments (ranibizumab, focal/grid laser photocoagulation, et al.) group (MD=-33.76; 95%CI: -45.53 ~ -21.99; *P*<0.001).

#### 3.4.3 Adverse events

**Figure 7:**
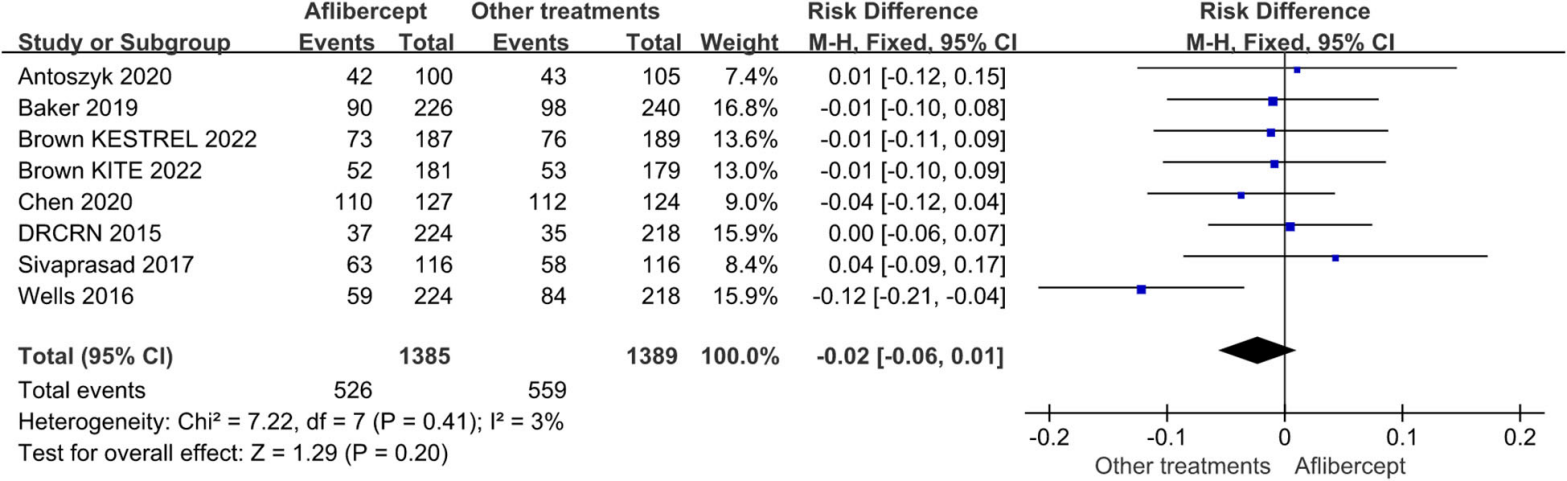
The adverse events in the aflibercept group and other treatments group.

Of the 16 RCTs involved, the overall incidence rates of ocular and non-ocular adverse events were similar across the treatment groups (**Figure 7**). Regarding the frequency or pattern of serious ocular adverse events, there was no significant difference between the aflibercept group and other treatments (ranibizumab, focal/grid laser photocoagulation, et al.) group (RD=-0.02; 95%CI: -0.06 ~0.01; *P*=0.41).

## 4 Discussion

In early 1976, the Diabetic Retinopathy Study group adopted PRP as the gold standard for the treatment of high-risk PDR eyes (25, 26). Since the ETDRS was first published in 1985, focal/grid laser photocoagulation has become the gold standard for the treatment of DME (27). Research on the Diabetic Retinopathy Clinical Research Network (DRCRnet) has confirmed that anti-VEGF drugs are not only effective alternative for PRP in patients with PDR but also as the first-line treatment for DME (28). It is very important for ophthalmologists and policymakers to compare the relative efficacy of DME or PDR treatment with the most reliable method.

Optical coherence tomography (OCT) is a noninvasive and easy-to-perform imaging tool that provides reliable and high-resolution imaging for the observation of retinal anatomy and quantification of the CST (29–31). With the help of OCT and the emergence of anti-VEGF drugs for patients with DR, clinical data suggested that anti-VEGF therapy can reduce macular edema and exudation, thus improving VA, reducing CST, and preventing further vision decline. Our findings are similar to those of previous studies from the viewpoint that anti-VEGF therapy is effective in patients with DME (32, 33). The therapeutic effects of these drugs are obviously superior to those of focal/grid laser photocoagulation separately.

To date, there are few meta-analyses comparing the expected clinical effects of aflibercept with other treatments (ranibizumab, focal/grid laser photocoagulation, PRP, pars plana vitrectomy, et al.) in the management of DME and PDR, especially the research on PDR.Based on this situation, we conducted a meta-analysis to compare the therapeutic effects of drug A with other treatment schemes. We evaluated16 RCTs published after 2015 in this meta-analysis, including 7125 patients who followed up 12 or 24 months. As reported in previous studies, among patients with visual impairment caused by DME, anti-VEGF monotherapy and combined focal/grid laser photocoagulation therapy provided better VA gain than focal/grid laser photocoagulation therapy separately (34). Although the short-term benefit of focal/grid laser photocoagulation combined with anti-VEGF therapy for DME patients was tiny in the DRCR.net Protocol I study, more than one-third of DME patients receiving anti-VEGF therapy delayed focal/grid laser photocoagulation therapy (35, 36). PRP has been the standard of care in the treatment of PDR for decades according to the DRS and the ETDRS (26). Both anti-VEGF therapy and focal/grid laser photocoagulation and PRP achieved remarkable anatomical and functional improvements during early treatment of DME and PDR respectively (37, 38). However, according to our results, for DME with long-term observation (a minimum of 12 months and up to 24 months), IAI had significantly better BCVA improvement than the focal/grid laser photocoagulation treatment (MD=13.30; 95%CI: 13.01~13.58; P<0.001) or other treatments(ranibizumab, focal/grid laser photocoagulation, PRP, et al.) (MD=1.10; 95%CI: 1.05~1.16; P<0.001). The visual improvements with IAI were primarily driven by patients with with poor baseline BCVA. In addition, IAI had significantly higher CST reduction than the focal/grid laser photocoagulation treatment (MD=-33.76; 95%CI: -45.53 ~ -21.99; *P*<0.001) or other treatments (ranibizumab, focal/grid laser photocoagulation, et al.) (MD=-33.76; 95%CI: -45.53 ~ -21.99; *P*<0.001). The increased response of patients with intractable DME or PDR may reflect the special pharmacological characteristics of aflibercept. This may be due to the fact that only aflibercept can inhibit both VEGF and placental growth factor (PGF), which are key factors leading to the pathogenesis of DME or PDR (39). More importantly, aflibercept has a faster association rate and a higher binding affinity for VEGF-A, VEGF-B, PlGF-1 and PlGF-2, resulting in accelerating a doubling of response rate (40).

The ability to achieve significant visual improvement with less frequent intravitreal injections and visits will be a valuable strategy for managing DME. In the VISTA and VIVID study, it was reported that the less frequent intravitreal injections schemes of aflibercept 2mg every 8 weeks (2q8) and 2mg every 4 weeks (2q4) can achieve similar effects in visual and anatomical results (12). In clinical practice, IAI 2q8 is also a good choice for working-age patients who have to miss work because of frequent visits. However, further studies are needed to determine the frequency of administration in patients with PDR.

Although anti-VEGF therapy can improve the visual and anatomical functions of patients with DR, focal/grid laser photocoagulation or PRP may still play an important role as an adjuvant therapy (41–43). PRP treatment was not a “one and done” procedure, and the addition of anti-VEGF drugs prevent DR progression and provide a “window period” for PRP. On the other hand, PRP can improve retinal oxygenation and decrease the drive for VEGF production by the retina, thus reducing the number of injections required and the burden of treatment. The combination of PRP and anti-VEGF therapy is acceptable in the real world. What’s more, according to the research of Protocol W, although there was no short-term vision benefit, early treatment with aflibercept can positively restore the anatomical structure of NPDR and reduced the risk of PDR or CI-DME with vision loss development in eyes with moderate to severe NPDR (44). Aflibercept may play a certain advantage in the management of DR with different severity.

Refractory vitreous hemorrhage and tractional retinal detachment may occur when PDR develops uncontrollably into advanced pathologies (45–47). For patients with recalcitrant DME, pars plana vitrectomy (PPV) can improve ocular anatomy (48, 49). Under these circumstances, PPV still plays a key role in the treatment of DR. Some studies have shown that anti-VEGF treatment before PPV can reduce intraoperative and postoperative hemorrhage and improve postoperative VA (50). On the positive side, in some cases requiring PPV, the combination of anti-VEGF therapy has a better curative effect. However, whether PPV has a wider effect than continuous anti-VEGF treatment has not yet been confirmed in RCTs.

The main unchangeable determinant of the development of diabetic retinopathy is the duration of diabetes (51). According to the standards for medical care for diabetes published by the American Diabetes Association in 2021, patients with type 1 diabetes should have a comprehensive ophthalmological examination within 5 years after diagnosis, and patients with type 2 diabetes should have their first fundus examination as soon as possible after diagnosis (52). A large-scale real-world research conducted by Chawla et al. found that the duration of diabetes was a strong predictor of the occurrence and development of DR. When the duration of type 2 diabetes reaches 9.4 ± 6.0 (mean ± SD) years, patients may have retinopathy. Therefore, we should always emphasize the early diagnosis and treatment of diabetic retinopathy in diabetic patients (53). It is worth noting that most people with diabetes in the RCTs we included are middle-aged and elderly people or working people. This kind of people are weak in physical examination consciousness or busy with work, and patients may delay the diagnosis of diabetes for various reasons, and their body are in a state of persistent hyperglycemia without knowing it. As a results, many patients with DR started their disease managements very late, or the medical treatment processes were irregular. Therefore, the scientific and standardized management of national health examination and disease prevention and control can not be ignored.

Studies have shown that the duration of action of anti-VEGF drugs varies among individual patients (32, 49). Moreover, even with appropriate treatment, repeated injections can increase the risk of infection, endophthalmitis, ocular inflammation, stroke, or myocardial infarction. The overall incidence of ocular and non-ocular adverse events in each treatment group was similar, and there was no significant difference between the aflibercept group and the other treatments group. It is a public knowledge that patients with diabetes have a high risk of cardiovascular comorbidities. In addition to diabetes, they are vulnerable to systemic complications. One study suggested that the increase in potential cerebrovascular accidents two years after treatment may be related to pro-epidermal growth factor therapy (54). Anti-VEGF therapy should be used cautiously in patients with myocardial infarction and stroke. Therefore, drug selection, injection frequency and interval, and necessary treatments should be adjusted according to the patient’s individual function and anatomical structure.

There are several limitations in this research. First, there may be deviations in the data collection, and only a small number of RCTs were included. In addition, with the rapid development of multi-mode imaging technology, methods for evaluating the prognosis of DR are more diversified. For example, with the addition of swept-source OCT, quantitative evaluation indices are more extensive, which will also affect our results. In future work, we will incorporate more indicators to quantitatively evaluate nonproliferative diabetic retinopathy and PDR. Additional OCTA parameters will be included in subsequent meta-analyses to improve the accuracy and robustness of the above conclusions and to provide better clinical guidance.

## 5 Conclusions

This meta-analysis showed that the advantages of IAI are obvious in the management of DME and PDR with poor baseline vision for long-term observation (a minimum of 12 months and up to 24 months). Applied IAI separately trended to be more effective than PRP separately with VA improvement for long-term observation. More parameters should be required to assess functional and anatomic outcomes.

## Author contributions

TL, XX, and CL designed the study and conducted data extraction and quality evaluation. XX and ZZ drafted and revised the manuscript. ZZ and MF conducted software analysis and data verification. WW and XX sorted data. XY generated the graphics and tables. YS searched literature. All authors contributed to the article and approved the submitted version.
